# Clinical use of biomarkers of survival in pulmonary fibrosis

**DOI:** 10.1186/1465-9921-11-89

**Published:** 2010-06-28

**Authors:** Michiel Thomeer, Jan C Grutters, Wim A Wuyts, Stijn Willems, Maurits G Demedts

**Affiliations:** 1 Department of Respiratory Medicine, Ziekenhuis Oost-Limburg, Genk, Belgium; 2 Respiratory Division, Universitaire Ziekenhuizen KULeuven, Leuven, Belgium; 3 Respiratory Division, St. Antonius Ziekenhuis, Nieuwegein, the Netherlands; 4 Divisie Hart & Longen, Universitair Medisch Centrum Utrecht, the Netherlands; 5 Laboratory of Pneumology, Katholieke Universiteit Leuven, Leuven, Belgium

## Abstract

**Background:**

Biologic predictors or biomarkers of survival in pulmonary fibrosis with a worse prognosis, more specifically in idiopathic pulmonary fibrosis would help the clinician in deciding whether or not to treat since treatment carries a potential risk for adverse events. These decisions are made easier if accurate and objective measurements of the patients' clinical status can predict the risk of progression to death.

**Method:**

A literature review is given on different biomarkers of survival in interstitial lung disease, mainly in IPF, since this disease has the worst prognosis.

**Conclusion:**

Serum biomarkers, and markers measured by medical imaging as HRCT, pertechnegas, DTPA en FDG-PET are not ready for clinical use to predict mortality in different forms of ILD. A baseline FVC, a change of FVC of more than 10%, and change in 6MWD are clinically helpful predictors of survival.

## Introduction

Interstitial lung diseases (ILD) [1] are a heterogeneous group of lung diseases that comprise more than 200 clinical pathological entities. Although clinically the different ILD have rather similar presentations with increasing shortness of breath, a restrictive lung function, impaired gas exchange and a widespread shadowing on the chest radiograph, they comprise a very wide spectrum of pathologies, clinical manifestations, and outcomes. Approximately two-thirds of ILD cases have no reported aetiology [2]. The remaining one-third is associated with or defined by various environmental or occupational factors including cigarette smoking, aspiration, certain drugs and radiation therapy [3,4]. Despite their acknowledged complexity, there is little evidence about the best management of ILD. Morbidity of the ILD themselves and adverse events of the available treatments may be high, with potentially serious consequences therefore for mismanagement. Improved survival and cure from different forms of ILD are dependent on a better understanding of the pathophysiology of the disease, its diagnostic accuracy in clinical practice, and an analysis of possible biomarkers which can guide the clinician in their treatment [5].

The clinical course of individual patients with ILD is variable and can manifest long periods of stability, a steady gradual decline, and/or periods of acute deterioration [6]. Some forms of ILD respond well to therapy, others are insensitive to high doses of anti-inflammatory drugs (e.g. dexamethasone) or immunosuppressive agents. Lung transplantation is an option for some patients, but many patients are too old or die on the waiting list [7]. One potential reason for the high mortality on the lung transplant waiting list is the variable course of the disease, which makes it difficult to predict outcome. Consequently, the identification of predictors of survival is critical for physicians and patients [8].

In the search for an effective therapy, biologic predictors of survival in pulmonary fibrosis with a worse prognosis, more specifically in IPF, have been extensively studied in the last 10 years. These surrogate endpoints for survival are so-called biologic markers or biomarkers. They can be subdivided into those markers measured in serum, those measured by lung function testing and those by imaging techniques. Before discussing the different results of the studies performed on predictors of survival, an introduction about what a biomarker is, is given.

## What is a biomarker ?

"A biomarker indicates a change in expression or state of a biologic measurement (e.g. concentration of a protein in serum, lung function measurement, amount of ground glass on HRCT, ...) at a given time point that correlates with the risk or progression of a disease, or with the susceptibility of the disease to a given treatment at a future time point [9]". Once a proposed biomarker has been validated, it is used to diagnose disease risk, presence of disease in an individual, or to tailor treatments for the disease in an individual [9]. In evaluating potential drug therapies, a biomarker may be used as a surrogate for a natural endpoint such as survival or irreversible morbidity [9]. If a treatment alters the biomarker, which has a direct connection to improved health, the biomarker serves as a surrogate endpoint for evaluating clinical benefit [9].

Use of such a biomarker as surrogate endpoint to assess a clinical endpoint (e.g. survival) has potential disadvantages. Biomarkers can be difficult to validate and require different levels of validation depending on their intended use [10]. If a biomarker is used to measure the success of a therapeutic intervention, the biomarker should reflect a direct effect of that intervention [10].

In summary, 4 pitfalls are present during the validation process of a biomarker as a diagnostic test [11]: (1) presence of a "spectrum bias", i.e. the study population on which the biomarker was validated has a different clinical spectrum (e.g. more advanced disease) than the population in whom the test has to be applied; (2) presence of a "selection bias", i.e. the test results of the validation study are related to test results (e.g. FVC < 90%); (3) presence of an "observer bias", i.e. observer (e.g. pathologist) is influenced by prior knowledge; and (4) presence of an "observer variability", i.e. the presence of variability in the interpretation of the results by the same observer (intra-observer variability) or by different observers (inter-observer variability).

## Serum biomarkers

At the end of the 1990s various serum markers were tested for their use in ILD, more specifically in IPF. Many markers have been studied and those with the most promise are surfactant proteins A and D (SP-A and SP-D), KL-6, and lactate dehydrogenase (LDH). In 2009 serum CC-chemokine ligand 18 (CCL-18) was presented as a potential biomarker in IPF [8]. Serum SP-A and SP-D are hydrophilic surfactant proteins produced and secreted by type II pneumocytes. Their concentration is elevated in certain inflammatory lung diseases, including IPF [12]. Why the concentrations of these proteins are elevated in these lung diseases is not precisely known. It is probably due to a combination of a loss of integrity of the epithelial barrier caused by lung injury and of an increased mass of type II cells due to hyperplasia [12]. Three studies tried to validate the use of these serum markers in an IPF population as a predictor for survival. The study of Takahashi et al. [13] concluded in a population of 52 IPF patients (mean follow up time 11.4 months for the subjects who died, more than 3 years for the survivors) that in the group of survivors (n = 10) the concentrations of SP-A and SP-D were within the normal range. In those subjects who died, around 25% also had protein concentrations within the normal range [13]. The second study by Greene et al. validated the use of SP-A and D in a population of 142 IPF patients as a predictor of survival. They used a Cox's proportional hazards model and found that an elevated concentration of SP-D (and not for SP-A) had a 56% elevated increase in death after adjusting for age, smoking status, TLC, FVC, FEV_1_, D_L_co and resting P_A-a _O_2 _[12]. The third study was recently published by Kinder et al [14]. It was found in 82 IPF patients that each increase of 49 ng/mL in baseline SP-A level was associated with a 3.3 fold increased risk of mortality in the first year after presentation. They observed no statistically significant association with serum SP-D and mortality [14]. In conclusion SP-A and D are promising biomarkers, but can not yet be used as a biomarkers for survival. When reviewing the studies, large standard deviations of the measured surfactant concentrations were found. This questions the reproducibility of the measurements.

In 1989, Kohno et al. discovered a compound named KL-6, a mucin-like high-molecular weight glycoprotein, which is expressed on type II alveolar pneumocytes [15]. Concentrations of KL-6 in serum and broncho-alveolar lavage fluid are elevated in different forms of ILD [15]. However, an elevated concentration of KL-6 is not specific for alveolitis in ILD, as this is also seen in breast cancer [16], non small cell lung cancer [17], colorectal cancer [18] and pulmonary tuberculosis [19]. Yokoyama et al. published a study of 14 IPF patients who received a predefined therapy of a weekly pulse of high dose corticosteroids for at least 3 weeks. The concentration of KL-6 was measured one week before and, 1 and 3 weeks after start of treatment. They found that a decrease in concentration of KL-6 was a good predictor of survival (n = 8) [20]. Different studies have been published indicating that the presence of alveolitis is correlated with an elevated concentration of KL-6 [21-23]. However, most of these studies have been performed with small number of subjects and therefore use of KL-6 as a biomarker for prognosis or response to therapy in ILD still needs to be validated with an unbiased and representative study population.

Some authors suggest that LDH is a potential marker of disease activity and of response to therapy in different forms of ILD [24-27]. Cytoplasmatic enzymes, like LDH, when present in the extracellular space are indicators of cell damage or cell death [25]. A case report of an IPF patient suggests a good correlation between level of serum LDH and response to therapy [28]. In contrast the study of Thomeer et al. found no correlation between survival and concentration or change in concentration of serum LDH. Our results can, however, be biased by the fact that an increase of LDH is also seen with intake of azathioprine, a drug that was given to all patients in this study. Serum LDH is a sensitive marker for cell injury, but is a non specific test since the concentration is elevated in different circumstances of cell injury caused by ischaemia, as by excess heat or cold, starvation, dehydration, injury, exposure to bacterial toxins, after ingestion of certain drugs, and from chemical poisonings [25]. Consequently, serum LDH is difficult to use as a valid biomarker of disease activity in ILD since its concentration is defined by many factors.

CCL18 is a CC-chemokine produced by human myeloid cells [8]. CCL18 is known to trigger a biological response in vitro in T cells, B cells, dentritic cells, hematopoietic progenitor cells, fibroblasts, and potentially, in monocytes/macrophages but not in neutrophiles [29]. It is constitutively expressed at high levels in lung and at low levels in some lymphoid tissues such as lymph nodes, thymus, and appendix [29]. Macrophages that are activated in a specific way, produce CCL18 and play a role in tissue repair processes such as wound healing and fibrosis [8]. CCL18 regulates collagen production by lung fibroblasts and is abundantly produced by alveolar macrophages in patients with IPF [8,30,31]. Prasse et al. set up a prospective study of 72 IPF patients, followed during 24 months with a pulmonary function every 6 months, but without a standardised treatment. They found that baseline serum CCL18 concentrations predicted change in TLC and FVC at the 6-month follow-up. In the group with high serum CCL18 concentrations a higher incidence of disease progression was seen. A cut-off value of 150 ng/ml calculated by ROC analysis, predicts mortality (sensitivity 83%; specificity 77%, area under the curve 0.80), with a hazard proportional ratio adjusted for age, sex, and baseline pulmonary function data of 8.0 [8]. One may conclude from this single study that CCL18 is the first biomarker that predicts mortality in IPF in such a clear way. However the process of measuring CCL18 concentration by ELISA has some pitfalls and its reproducibility and internal validity are of concern. Prasse et al. suggest that a standardization of the entire procedure from drawing blood over freezing and thawing to ELISA measurement is mandatory to overcome this concern [8]. The findings of Prasse et al. induce important questions regarding the pathogenesis or the search for therapy of IPF. Is CCL18, that stimulates lung fibroblasts to produce collagen, the key to a potential new treatment in IPF [29]? Is the lack of an animal model for lung fibrosis explained by the fact that a rodent counterpart for human CCL18 does not exist [29]? Further studies are needed to elucidate these questions, and more precisely to discover a CCL18 receptor, which is still not found [29].

## Physiologic parameters as biomarker

### Pulmonary function

ILD are usually characterised by a restrictive lung function, i.e. a reduction in lung volumes with preserved Tiffeneau index (or FEV_1_/FVC ratio) together with a reduction in D_L_co. However, in early disease, lung volumes and D_L_co may be within the normal range [4]. Furthermore, in sarcoidosis and in Histiocytosis X evidence of airflow obstruction is also found in more than a quarter to half the patients in some studies [4]. Lung volumes may be relatively preserved in smokers with IPF possibly due to coexisting emphysema although the Tiffeneau index remains normal. This suggests that the using a restrictive lung function as an exclusive diagnostic biomarker for ILD is neither sensitive nor specific enough [4].

Serial lung function testing is used to monitor the clinical course of the disease [4]. VC and D_L_co are the most used and the simplest indicators to measure change in ILD [4]. There is, however, little agreement about how frequently these lung function measurements must be obtained in the follow up of the different forms of ILD. This is partly because the clinical course of the different ILD shows a wide variation, from acute alveolitis due to amiodarone to chronic fibrosis by scleroderma.

Different studies have addressed the question if lung function variables can be used to predict survival. A study of our group presented the survival rates between the most common forms of ILD, with a mean survival after 5 yrs of 91.6% in SARC, 84.1% in HP, 69.7% in CTD, 35.4% in IPF, 85.5% in other IIP and 69.5% in undefined forms of lung fibrosis [32]. A Cox regression analysis shows that an age of less than 66 year, a diagnosis of an ILD that is not an IPF, a VC of more than 63% predicted and a % macrophages of less than 63% in BALF is indicative in this model of a lower mortality risk. D_L_co was not found as an independent variable for survival.

The spinoff of the IFIGENIA trial aimed to quantify the risk for mortality in 155 IPF patients after 4 years of the date of inclusion in the IFIGENIA study [33]. The study subjects were followed with measurements of VC, TLC and D_L_co at baseline, month 6 and month 12 after inclusion. A TLC > 62% predicted (HR 0.49; 95%CI 0.30-0.81) and a D_L_co > 43% (HR 0.37; 95%CI 0.23-0.61) at baseline were found to influence rate of survival by a Cox's regression analysis. Changes in VC or D_L_co over 6 or 12 months were not found to be independent variables of survival.

Martinez et al. analysed retrospectively data from the placebo group (168 IPF patients with mild to moderate disease) of a trial evaluating interferon gamma as treatment for IPF [6,34]. At 12 week intervals D_L_co and VC were measured over a median period of 76 weeks. Twenty one percent died, of which IPF (89%) was the primary cause of death [6]. In patients with an IPF-related death, 15 (47%) deaths were categorised as acute or abrupt and 16 deaths (50%) were considered subacute. For patients who survived to week 72, the mean percentage predicted FVC decreased from 64.5% (SD 11.1%) to 61.0% (SD 14.1%). The mean percentage predicted decreased from 37.8% (SD 11.1%) to 37.0% (SD 19.9%) [6]. For patients who died during this trial, a general trend toward a decrease in FVC and D_L_co was observed, although significant intra-patient variability occurred over time [6]. Martinez et al. concluded that the clinical course of patients with mild to moderate IPF was characterised by minimal physiologic deterioration as measured by FVC and D_L_co over a period of 76 weeks [6].

Flaherty et al. examined retrospectively 80 patients with IPF and 29 patients with NSIP [35]. They found that in a multivariate Cox proportional hazards model controlling for histopathologic diagnosis (UIP versus NSIP), gender, smoking history, baseline FVC, and 6 month change in FVC, a decrease in FVC of more than 10% remained an independent risk factor for mortality (HR 2.47; 95%CI 1.29-4.73) [35].

Various other investigators have also suggested that a decreased FVC at baseline identifies patients at subsequent risk of mortality [36-40]. In addition, some suggested that a decrease in FVC of 10% or more after 1 year predicts mortality in patients with IPF [41]. Spirometric assessment is particularly valuable as its measurement is standardised [43] and the variability in FVC is well defined among normal subjects and patients with pulmonary disease [42,43]. Despite these standards, a variability in the FVC measurements of patients with IPF over time is noted [44,45]. As a result, a wide variety of thresholds for change in FVC is used, including changes ranging from 10 to 15% in FVC [35,39,41,46-48].

Flaherty et al. demonstrated that the change in D_L_co over 6 months of follow-up has limited prognostic value and this measurement was not found to add independent predictive value for mortality in IPF [35]. Although standards are presented for its measurement [49,50], the D_L_co varies to an even greater extent than FVC and clinically significant changes are believed to be more than 20% [35,41,46,47,51]. As such, a survival advantage was noted by one group in patients with an improved or unchanged D_L_co compared with those experiencing a decrease of 20% or more after 1 year of therapy [35].

The question remains whether FVC and D_L_co are useful as surrogate markers for survival in trials searching for therapeutic strategy in IPF? This question is critical since change from baseline to a specified time point of these physiologic variables has been used either or both as primary endpoint in the most recent therapeutic trials in IPF [52].

The INSPIRE trial, the largest trial ever, included 1373 IPF patients, and concluded that no difference is seen in survival between those treated with interferon gamma and those with placebo [53]. Consistent with the findings as described above, the INSPIRE investigators noted negligible changes in mean values of FVC and D_L_co during 77 weeks treatment with either interferon gamma or placebo [53].

With the available evidence, it is difficult to conclude that a decrease or a change over time in FVC or D_L_co are valid biomarkers for survival, because the results of above mentioned trials are confusing. Is survival itself the only valid endpoint to be used in therapeutic trials? IPF is a rare disease, and if investigators decide only to use survival as primary endpoint, trials will be difficult to set up and manage to a successful conclusion. Taking the INSPIRE study as an example, the investigators calculated at the onset of the trial that to have 90% statistical power to detect a treatment effect equivalent to a 50% reduction in 3-years, about 600 patients were needed to achieve the targeted number of deaths within the planned duration of the study (77 weeks). Two interim analyses of this trial increased the number to 1200 patients. The economic and logistic efforts (worldwide participation of 82 centres) to bring this to a successful conclusion are huge. Efforts not every investigator or pharmaceutical company can afford [54].

### Six minute walking distance

Different studies have evaluated the correlation between 6 minute walking distance (6 MWD) or change in walking distance and survival [55-61]. Most of these studies are characterised by small sample size, and therefore yielded inconsistent results related to survival. Only one study, recently presented as an abstract, found in a post hoc analysis of the INSPIRE trial of 822 IPF patients, that a 24 week change in 6 MWD was highly predictive to mortality: a 24 week decrement of > 50 m was associated with a 4.3 fold increase in one-year mortality, a decrement between 26-50 m an increase of 3.6 [62].

## Biomarkers measured by medical imaging

Two types of medical imaging are discussed. HRCT and nuclear imaging including imaging that measures the alveolar capillary membrane permeability of the lung by use of radioactive isotopes, pertechnegas and ^99m^Tc-diethylenetriamine pentacetate (DTPA), and positron emission tomography (PET).

It is nowadays unthinkable not to use HRCT in the diagnosis of ILD. The added value of HRCT depends upon its ability to increase confidence of a specific diagnosis, to alter patient management and, if possible, to influence outcome [4]. Research on the ability of HRCT scans to differentiate between active and inactive disease has been mainly confined to IPF and ILD associated with systemic sclerosis [4]. There is evidence that a predominant ground glass pattern is more likely to represent active inflammatory disease and to respond to appropriate therapy, particularly in fibrosing alveolitis, EAA, and desquamative interstitial pneumonia [63-65]. It is still unproven that a ground glass pattern precedes a reticular or honeycomb pattern, although this seems likely [4]. Not all ground glass change indicates cellular inflammation, however, as fine intralobular fibrosis may be indistinguishable from a cellular infiltrate on HRCT scans [64,66]. The association of a ground glass pattern with traction bronchiectasis or bronchiolectasis is likely to indicate some associated fibrosis, whereas ground glass change without traction bronchiectasis usually indicates active inflammation [66]. Reticular and honeycomb patterns on HRCT scans correlate well with histological evidence of fibrosis [67,68].

Can HRCT predict response to therapy in IPF? Gay et al. set up a study with 38 biopsy proven IPF patients. The study patients received 1 mg/kg prednisone daily during 3 months. The HRCT before treatment was scored (score from 0 to 5 for each lobe) for ground glass and fibrosis by 4 radiologists independently. They demonstrated that a fibrosis score of 2 or more has a 80% sensitivity and 85% specificity in predicting survival [69]. From this study it is not clear how many drop outs were present during the survival follow up and how long the time of follow up was, two factors that can induce a possible bias in their results. A spin off trial of the IFIGENIA study used the same scoring method proposed by Gay et al. The HRCT was scored before start of a predefined treatment of azathioprine and prednisone and at 6 and 12 months of treatment. The HRCT scores for fibrosis and ground glass were assessed by 3 radiologists independently. In this study 155 IPF patients had a median follow up of 2.5 yrs (SD 1.8). Only the fibrosis score at baseline was predictive for survival (HR 1.58 95% CI 1.15-2.17), whereas ground glass score or changes in ground glass score or fibrosis score over 6 and 12 months were not predictive for survival. A HRCT fibrosis score of more than 2 had a relative risk for death of 2.31 (95%CI 1.40-3.80). However the area under the curve for the fibrosis score was only 0.61 (95%CI 0.52-0.70), which means that the score had only a moderate to low sensitivity and specificity for survival.

The ILD are characterised by an acute or chronic inflammation of the interstitium, also called the alveolar capillary membrane [70]. A possible way to measure the alveolar capillary membrane permeability is by radionuclide aerosol lung imaging. The rate of the clearance of the aerosol is inversely related to the integrity of the alveolar capillary barrier [71-73]. Pertechnegas and DTPA have been studied as disease activity measure in different forms of interstial lung diseases, but, as far as we known, no studies has correlated rate of lung clearance with survival [73,74].

Positron emission tomography (PET) imaging has recently emerged on the scene of biomarkers of ILD. A number of studies has suggested that ^18^F-FDG PET imaging may serve as a sensitive tool for the evaluation of disease activity in sarcoidosis, with higher sensitivity and interobserver agreement compared to the classical Gallium scintigraphy [75-77]. The potential value of ^18^F-FDG PET as a biomarker for disease activity in other ILD is less clear. In IPF the magnitude of ^18^F-FDG uptake in the lungs is usually low. As ^18^F-FDG is thought to assess the inflammatory burden and not the fibrosis, the finding of relatively low SUV in IPF can be regarded as confirmative for the concept that inflammation does not play a major role in the pathogenesis of this disease. No studies are present that correlates rate of ^18^F-FDG uptake with survival in specific forms of interstitial lung diseases.

## Conclusion

Interstitial lung diseases are a diverse and complex collection of parenchymal lung diseases that vary widely in aetiology, histopathology, clinical radiological presentation, and clinical course [78]. There is an urgent need for biomarkers or markers of disease activity that would help the clinician in deciding whether or not to treat since treatment carries a potential risk for adverse events. These decisions are made easier if accurate and objective measurements of the patients' clinical status can predict the risk of progression to death. Serum biomarkers, and markers measured by medical imaging as HRCT, pertechnegas, DTPA en FDG-PET are not ready for clinical use to predict mortality in different forms of ILD. A baseline FVC, a change of FVC of more than 10%, and a decrement of more than 25 m in 6 MWD are predictors of survival. Measurements of FVC and 6 MWD are clinically easy to do, results are measured in no time and pose minimal effort for the patient.

## Competing interests

The authors declare that they have no competing interests.

## Authors' contributions

All authors wrote and revised the manuscript, and approved the final version
